# Editorial: SARS-CoV-2 variants, B lymphocytes, and autoreactivity

**DOI:** 10.3389/fimmu.2022.1125732

**Published:** 2023-01-10

**Authors:** Marko Z. Radic, Moncef Zouali

**Affiliations:** ^1^University of Tennessee, College of Medicine, Memphis, TN, United States; ^2^Graduate Institute of Biomedical Sciences, China Medical University, Taichung City, Taiwan

**Keywords:** B cell, COVID - 19, memory, antibody repertoire analysis, variable gene selection

Since its identification in 2019, SARS-CoV-2 has caused the ongoing COVID-19 pandemic that resulted in over six 6 million deaths worldwide. Similar to other Coronaviruses, infections with SARS-CoV-2 progresses in a multistep manner that involves cleavage and rearrangement of the surface spike protein (S) that uses the receptor binding domain (RBD) to engage angiotensin-converting enzyme 2 (ACE2) on target cells (1). The rapid evolution of SARS-CoV-2 and the emergence of variants of concern (VOC), such as the omicron (B.1.1.529) mutant, represent challenges for the immune system, with implications for designing potent vaccines and developing therapeutic antibodies. Articles in this Research Topic shine a light on the B cell responses mounted by the immune system to cope with infection to, and vaccination against SARS-CoV-2.

## Antigenic cartography and dynamic changes

SARS-CoV-2 variants exhibit characteristic mutations in the S protein, including the RBD, which represents an immunodominant part of the S protein and is targeted by a large proportion of neutralizing antibodies (2, 3). Several S protein-based serological assays have been developed to profile the anti-SARS-CoV-2 humoral immune response and to analyze its dynamic changes, including ELISA assays, peptide microarrays, and antibody binding epitope mapping (Chen et al.). More recently, flow cytometric approaches have been introduced using cells expressing native S proteins in the same orientation and a glycosylation pattern similar to that found on the viral membrane. In this collection, Vesper et al. describe a flow cytometric assay that, in combination with a color-coded barcoding method, allow comparison of binding of S proteins or RBD mutants to soluble ACE2-Ig molecules, or antibodies of different classes in the sera of vaccinated or infected subjects. This new assay should be valuable for further evaluations of the humoral response to SARS CoV-2 variants.

Understanding the mechanism underlying viral evasion from the immune response is important for designing effective vaccines and therapeutics. Thus, the Omicron variant (B.1.1.529.1) exhibits 34 mutations in its S protein, including 15 changes in the RBD, compared with the original Wuhan SARS-CoV-2 strain reported in 2019. These mutations could facilitate viral internalization through binding to ACE2 on target cells and promote the increased immune evasion potential of VOC. Comparison of the sequences of SARS-CoV-2 proteins from multiple ancestral strains, including Omicron variants (BA.1, BA2, BA3, BA.4, BA.5, BQ.1 and BBX.1), and the previously circulating Alpha, Beta, Gamma, and Delta strains, revealed that Omicron and it’s sub-variants exhibit a bias toward Asparagine to Lysine transitions within the S protein (Boer et al.). This mutation could lead to conformational changes, thereby potentially reducing the activity of neutralizing antibodies in infected subjects.

## Mining antibody genes

Initial surveys of antibody repertoire in COVID-19 patients noted a polyclonal response associated with high numbers of circulating plasmablasts and low-levels of somatic mutations (4). Further analysis of RBD-specific antibodies from convalescent subjects revealed that the acquisition of somatic mutations and affinity maturation could impart protection against diversifying SARS-CoV-2 variants (5). In this collection, two cohorts of not previously infected patients were used to probe somatic hypermutation at distinct points after vaccination (Paschold et al.). After the priming vaccinations, B lineage evolution and somatic hypermutation were low. With booster vaccinations, antigen-experienced B cell clones were mobilized to further rapid somatic hypermutation, suggesting that affinity maturation may account for the increased protection of booster injections against SARS-CoV-2 variants, such as the Omicron variant B.1.1.529.

Taking a different research angle, Stewart et al. compared the antibody repertoire during pandemic, epidemic and endemic viral disease by tracking the V-D-J sequences in the context of antibody subclasses in B cell responses to COVID-19, Ebola virus disease survivors from West Africa and the UK, volunteers challenged with Respiratory Syncytial Virus, and samples from healthy donors. They report that, while features of B cell responses are specific for particular infections, the immunoglobulin repertoire can exhibit similarities across very different diseases, such as a general increase of IGHV4-39 gene used in response to SARS-CoV-2 and Ebola virus infection.

## B lymphocyte population dynamics

In an in-depth commentary, Rossi et al. present an overview of IgM memory B cells with a focus on human secondary lymphoid structures in the spleen and list evidence for the protective role of IgM and IgA antibodies to SARS-CoV-2. Most notably, the authors cite the observation that patients, who were infected with SARS-CoV-2 but showed reduced or depleted levels of the IgM memory B cell subset, experienced more severe or fatal infections. The authors thus argue for the benefit of a close assessment of the immune status in newly infected individuals, such that prophylactic or therapeutic measures could be administered in a timely manner.

Taking a closer look at the risk of administering the B cell depleting anti-CD20 monoclonal antibody, Rituximab, as a therapy for the autoimmune disease rheumatoid arthritis, to patients, who also required protection from SARS-CoV-2 by standard mRNA vaccination against the virus, Stefanski et al. observed that the efficacy of a third vaccine dose was preserved, if Rituximab was given 1-2 months after the second dose of the vaccine. The conclusion was reached that generation of anti-SARS-CoV-2 memory B cells, protective antibodies, and spike-specific CD4 T cells, prior to CD20 B cell depletion, allows further boost of anti-viral responses and elicits a particularly strong IgA B cell population.

### SARS-CoV-2 infection and autoreactive B cells

The parallel observations that inflammatory cytokines, such as TNF−α, IL-1β and IFN-γ, favor the development of so-called double-negative CD27- IgD- (DN) B cells, a likely precursor population driving the production of autoantibodies in lupus, and the separate finding that SARS-CoV-2 infection may promote the expression of autoantibodies, prompted Castleman et al. to examine the relative abundance of DN B cells in COVID-19. The original discoveries reported in this paper included the diminished frequencies of DN1 B cells and elevated numbers of DN2 and DN3 B cells in severe COVID-19. Along with cytokine imbalances induced by the viral infection, the authors observed a notable expansion of DN3 B cells and the appearance of autoreactive antibodies within the pro-inflammatory milieu.

The induction of autoreactivities in a set of 31 individuals exposed to SARS-CoV-2 in the healthcare setting was also examined by Moody et al. The authors noted the presence of a set of antibodies to 11 autoantigens. Strikingly, the analysis, using an autoantigen array approach, identified the autoreactivity to calprotectin, a complex of the mammalian proteins S100A8 and S100A9, in one fourth of the analyzed plasma samples that persisted for eight months following the SARS-CoV-2 infection and correlating with complete recovery from the viral infection.

### Conclusions

In sum, the articles presented in this Research Topic represent a valuable set of diverse and innovative research that has been sparked by the COVID-19 pandemic. With determination and vigorous collaborative efforts, the scientific community is responding to the challenge and yielded numerous new insights into the pathogenesis of SARS-CoV-2 infections and immune responses, including humoral responses, alterations in B lymphocyte population dynamics, and effects on antibody repertoire profiles. Many of the newly emerging concepts will retain lasting impact, long beyond the current health emergency.

## Author contributions

Both authors listed have made a substantial, direct, and intellectual contribution to the work and approved it for publication.
